# African ancestry of New World, *Bemisia tabaci*-whitefly species

**DOI:** 10.1038/s41598-018-20956-3

**Published:** 2018-02-09

**Authors:** Habibu Mugerwa, Susan Seal, Hua-Ling Wang, Mitulkumar V. Patel, Richard Kabaalu, Christopher A. Omongo, Titus Alicai, Fred Tairo, Joseph Ndunguru, Peter Sseruwagi, John Colvin

**Affiliations:** 1 Natural Resources Institute, University of Greenwich, Central Avenue, Chatham Maritime, Kent, ME4 4TB UK; 2 Root Crops Programme, National Crops Resources Research Institute, P. O. Box, 7084 Kampala, Uganda; 3 Biotechnology Department, Mikocheni Agricultural Research Institute, P.O. Box, 6226 Dar es Salaam, Tanzania

## Abstract

*Bemisia tabaci* whitefly species are some of the world’s most devastating agricultural pests and plant-virus disease vectors. Elucidation of the phylogenetic relationships in the group is the basis for understanding their evolution, biogeography, gene-functions and development of novel control technologies. We report here the discovery of five new Sub-Saharan Africa (SSA) *B*. *tabaci* putative species, using the partial mitochondrial cytochrome oxidase 1 gene: SSA9, SSA10, SSA11, SSA12 and SSA13. Two of them, SSA10 and SSA11 clustered with the New World species and shared 84.8‒86.5% sequence identities. SSA10 and SSA11 provide new evidence for a close evolutionary link between the Old and New World species. Re-analysis of the evolutionary history of *B. tabaci* species group indicates that the new African species (SSA10 and SSA11) diverged from the New World clade *c*. 25 million years ago. The new putative species enable us to: (i) re-evaluate current models of *B. tabaci* evolution, (ii) recognise increased diversity within this cryptic species group and (iii) re-estimate divergence dates in evolutionary time.

## Introduction

*Bemisia tabaci* species are phloem-feeding insects that damage a wide range of crops, including beans, cassava, cotton, cucurbits, potato, sunflower and tomato^1,2^. This group of cryptic species also vector more than 200 plant viruses^3^ that cause a wide range of plant diseases associated with devastating economic losses to many agricultural crops worldwide^1,4,5^. To date, 39 morphologically indistinguishable, but genetically diverse species have been reported and they differ greatly in their biological characteristics^6–9^ including: host-plant range^10,11^, inducement of phytotoxic disorders^4,12^, resistance to insecticides^13,14^, invasiveness^4,15^ and specificity of begomovirus transmission^3,16^. For example, estimated economic losses attributed to one putative species, the ‘Middle East-Asia Minor 1′ (MEAM1) alone are US$714 million annually^17^.

*Bemisia tabaci* species are present across the globe between 30 °N and 30 °S^15^. In Africa, the *B. tabaci* species Sub-Saharan Africa 1‒5 (SSA1‒5), Uganda 3 (herein named Sub-Saharan Africa 6), Africa (named Sub-Saharan Africa 7), East Africa 1, Mediterranean (MED), Middle-East Asia Minor 1 (MEAM1), and Indian Ocean (IO) colonise important agricultural crops such as cassava, beans, sweetpotato, as well as several weed species^1,18–22^. The *B. tabaci* species that colonise cassava, specifically SSA1 and SSA2 vector two economically important viruses: cassava mosaic begomoviruses (CMBs) and cassava brown streak ipomoviruses (CBSI)^23,24^. The two whitefly species have been associated with significantly high whitefly abundance; SSA2 in the 1990s^1^ and SSA1 more recently^19,21^.

Genetic diversity of the *B. tabaci* group has been studied previously using various molecular markers^2,25,26^ including 16 S rDNA^25^, mitochondrial cytochrome oxidase 1 (*mtCO1*)^25^ and ribosomal internal transcribed spacer 1 (*ITS1*) sequences^27,28^. The most commonly used marker is a partial fragment of the *mtCO1* gene that has been used to establish the phylogeographic distribution of the group^1,2,25,26^. Amplification of the target *mtCO1* region has generally used a primer set (MT10/C1-J-2195 and MT12/TL2-N-3014)^29^, but despite its widespread use, problems with this primer set for the amplification of some *B. tabaci* DNAs have been reported^30^.

The geographical origin and distribution of the different species within the *B. tabaci* group has been investigated^2,25,26^ and Sub-Saharan Africa was inferred to be the likely centre of origin^26^. Frohlich *et al*.^25^ analysed a representative collection of *B. tabaci* from all around the world and identified two main groups: Old World and New World. The Old World group was further separated into the Indian subcontinent, equatorial Africa and Sahel-region groups^25^. Subsequent studies showed a similar geographical distribution pattern of *B. tabaci* species^1,28,31^. For example, New World species were identified in the Americas^4,32,33^, while the Asian species (Asia I–IV, Japan, China 1‒3)^7,34^ and SSA1‒6^20,21,35^ species were identified in Asia and Africa, respectively. Apart from MEAM1 and MED, *B. tabaci* species still occupy distinct geographical regions across the globe. The worldwide distribution of the MEAM1 and MED species has clearly resulted from recent introductions, through the movement of plant-material by humans, combined with the ability of these species to invade new regions and displace indigenous species^4,15,31^.

Since the report by Frohlich *et al*.^25^, additional *B*. *tabaci* genetic groups have been discovered in Sub-Saharan Africa that show the evolutionary importance of the region. These include a single specimen from Sudan (EU760727) that is probably a recent introduction from America, because it clusters within the New World clade (Supplementary Fig. S1), from Cameroon (EU760739; named Sub-Saharan Africa 7) that is ancestral to the Australia-Asia clade (Supplementary Fig. S1)^22,36^ and from Morocco (HE863764 and HE863760) that groups next to the Italy clade^37^. The single Sudanese specimen (EU760727) is not ancestral and so, prior to the discovery of the new species reported here, there was no strong evidence for an evolutionary link between African and New World species.

The discovery of a close link between African and New World *B. tabaci* requires a re-evaluation of the molecular dating evidence for the evolutionary divergence of *B. tabaci*. Previous analyses have suggested that the current bio-geographical distribution of *B. tabaci* species was due to the breakup of Gondwanaland and subsequent plate tectonic movements^36^. Western Gondwanaland was believed to have separated into South America and Africa from 120–84 mya^38^. About 95 ± 5 mya, Australia broke away from Antarctica, while India broke away from Madagascar and drifted north to collide with Asia^38–40^.

Evolutionary divergence dates for *B. tabaci* species remain controversial^36,41^, however, Campbell *et al*.^42^, Boykin *et al*.^36^ and Santos-Garcia *et al*.^41^ estimated, based on DNA sequences and fossil material^43^, that the genus *Bemisia* diverged from the other whiteflies approximately 90–87 million years ago (mya) when South America separated from Africa^38,44^. Boykin *et al*.^36^ estimated that the MEAM1 and MED species of the *B. tabaci* species complex diverged approximately 13 mya, while Santos-Garcia *et al*.^41^ estimated that MEAM1 and MED diverged approximately 2.9–0.4 mya. These estimates are inconsistent, but all agree that the evolutionary changes pre-date the advent of agriculture by millions of years. Here, we add the newly discovered species to published data and re-analyse the world-wide phylogenetic relationships and evolutionary history of the *B. tabaci* group of species.

## Results

### New primer design

**Figure 1:**
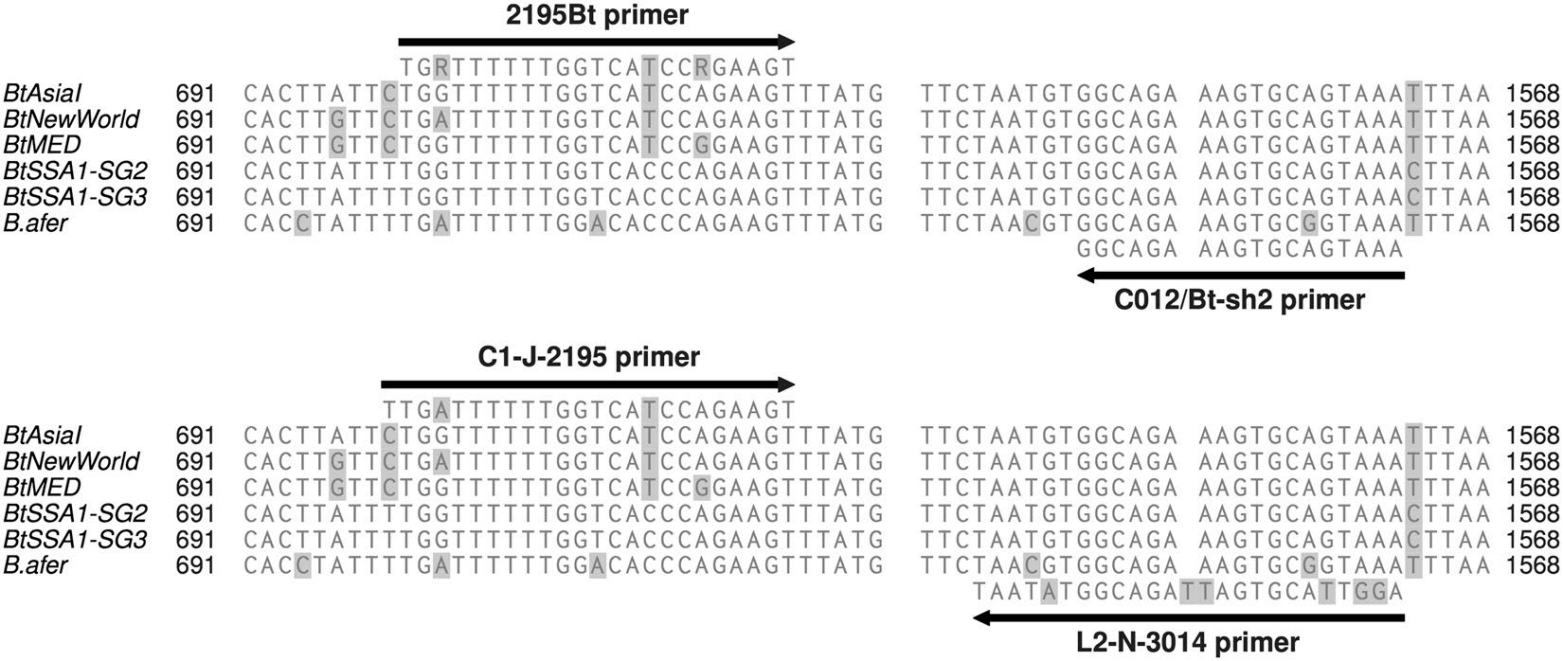
Alignment of partial *mtCO1* gene sequences of four *Bemisia tabaci* species and *Bemisia afer* showing the positions of the new (2195Bt and C012/Bt-sh2) and old (MT10/C1-J-2195 and MT12/TL2-N-3014) primer sets.

**Figure 2:**
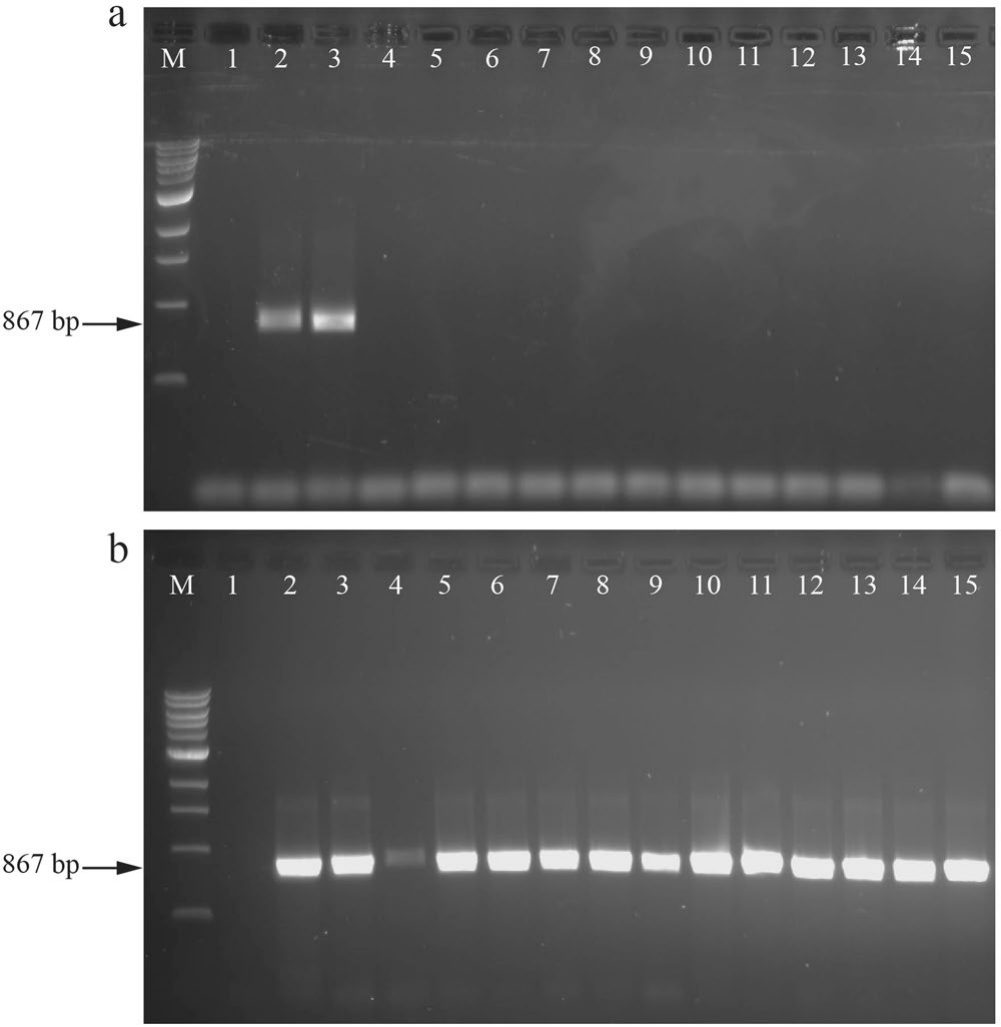
A comparison of the performance of the old (**a**) [MT10/C1-J-2195:MT12/TL2-N-3014] - Lane 1, negative control (no DNA template); lane 2 and 3, positive (whitefly DNA) controls; lane 4–15, whiteflies collected from mint (lanes 4–7), sesame (lanes 8–11) and sunflower (lanes 12–15) and new (**b**) [2196Bt:CO12/Bt-sh2] primer set with successful amplification using the same order of specimens. M, 1Kb plus molecular weight marker (BIOLABS, UK).

Total DNAs prepared from some of the *B. tabaci* specimen collected in our study were not amplified with the primer pair that has been used most frequently for this purpose to generate *B. tabaci mtCO1* partial sequences. This pair has a forward primer (MT10/C1-J-2195) designed to target insects in general, while the reverse primer (MT12/TL2-N-3014) targets gerrids, weevils, mosquitoes, flies and Lepidoptera^29^. To investigate why amplification was not occurring, these primer sequences were aligned with complete *mtCO1* sequences of *B. tabaci* and *B. afer* present in Genbank. Figure 1 shows the alignment and reveals that the above primers have several mismatches. This necessitated the development of a new degenerate primer set (2195Bt and C012/Bt-sh2) with improved specificity for *B. tabaci* and *B.afer* (Figs 1 and 2). The new primer set (2195Bt and C012/Bt-sh2) amplified all the specimens, including those that failed with the old primer set (Fig. 2).

**Figure 3:**
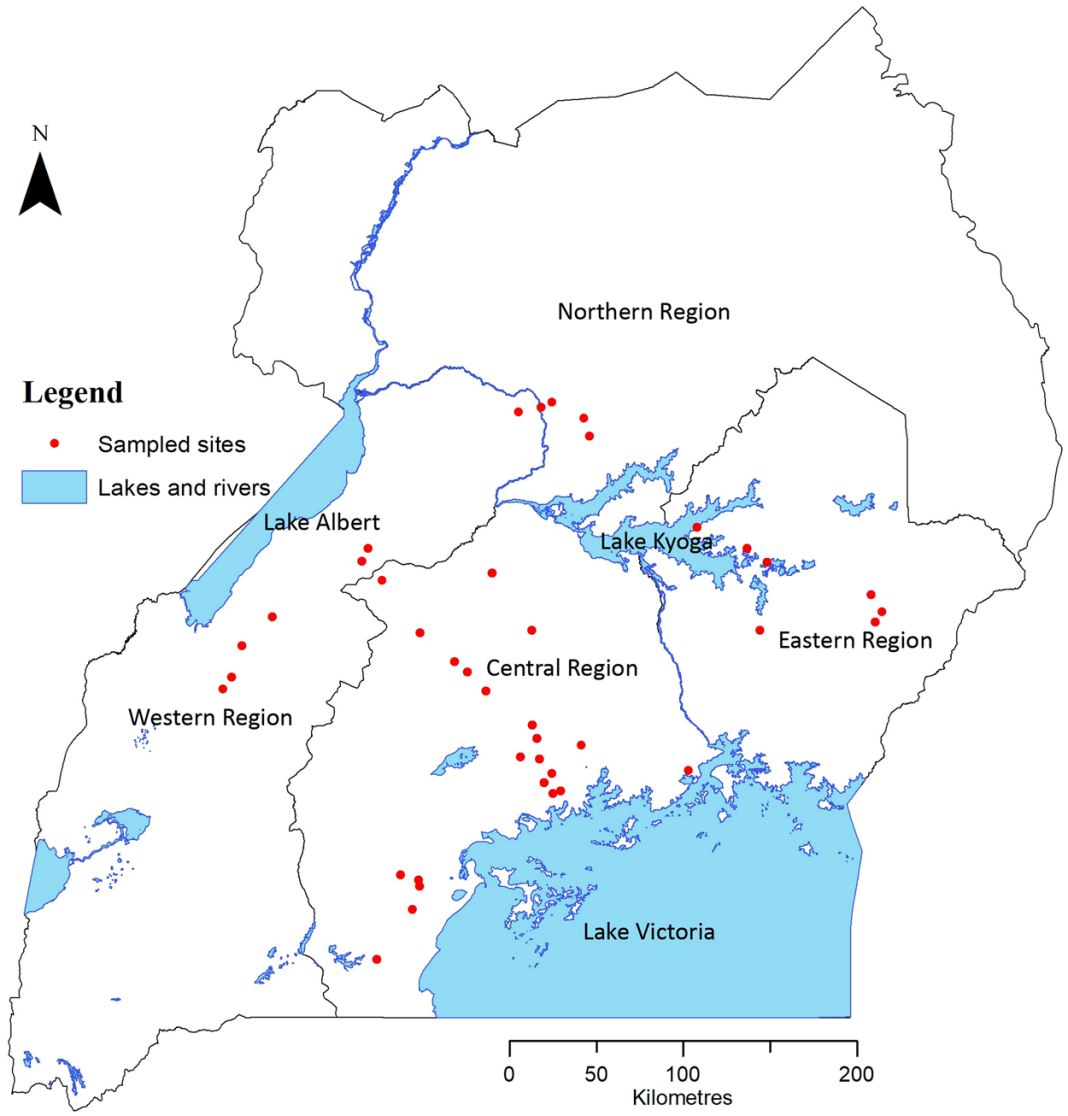
The locations (red circles) in Uganda where whitefly specimens were collected during August–November 2013.

To investigate the genetic diversity of *B. tabaci* species present in Uganda, adult whiteflies were collected from cassava and five weed species occurring commonly around cassava fields (Table 1 and Supplementary Dataset) in 26 districts (Fig. 3). The new degenerate primer set was used to generate a total of 121 partial (867 bp) *mtCO1* sequences (GenBank accession numbers KX570749–KX570869) that were used for species determination against the reference *B. tabaci* species^2,45^.

**Table 1:** Sample numbers of *B. tabaci* and non-*tabaci* species collected from cassava and weed species in Uganda in 2013.

**Plant/Whitefly species**	SSA1	SSA2	SSA6	SSA9	SSA10	SSA11	SSA12	SSA13	MEAM1	MEAM2	MED	IO	*B*. Uganda1	**Total**
*Manihot esculenta* (Cassava)	0	0	0	1	8	0	0	0	0	0	0	0	0	9
*Pavonia urens*	1	1	3	1	0	3	0	0	1	5	12	0	1	27
*Euphorbia heterophylla*	8	1	0	0	1	0	0	0	0	0	0	3	3	16
*Cleome gynandra*	2	0	0	0	0	0	0	0	0	0	9	0	3	14
*Vernonia amygdalina*	3	0	2	0	1	0	0	0	0	3	7	0	2	18
*Commelina benghalensis*	5	2	0	1	1	0	18	2	0	0	4	0	4	37

Putative species were assigned based on their partial *mtCO1* sequences according to Dinsdale *et al*. (2010). The abbreviations for the whitefly species are as follows: SSA is sub-Saharan Africa, MEAM is Middle East-Asia Minor, MED is Mediterranean, IO is Indian Ocean and *B*. Uganda1 is *Bemisia* Uganda 1.

### Phylogenetic analysis

**Figure 4:**
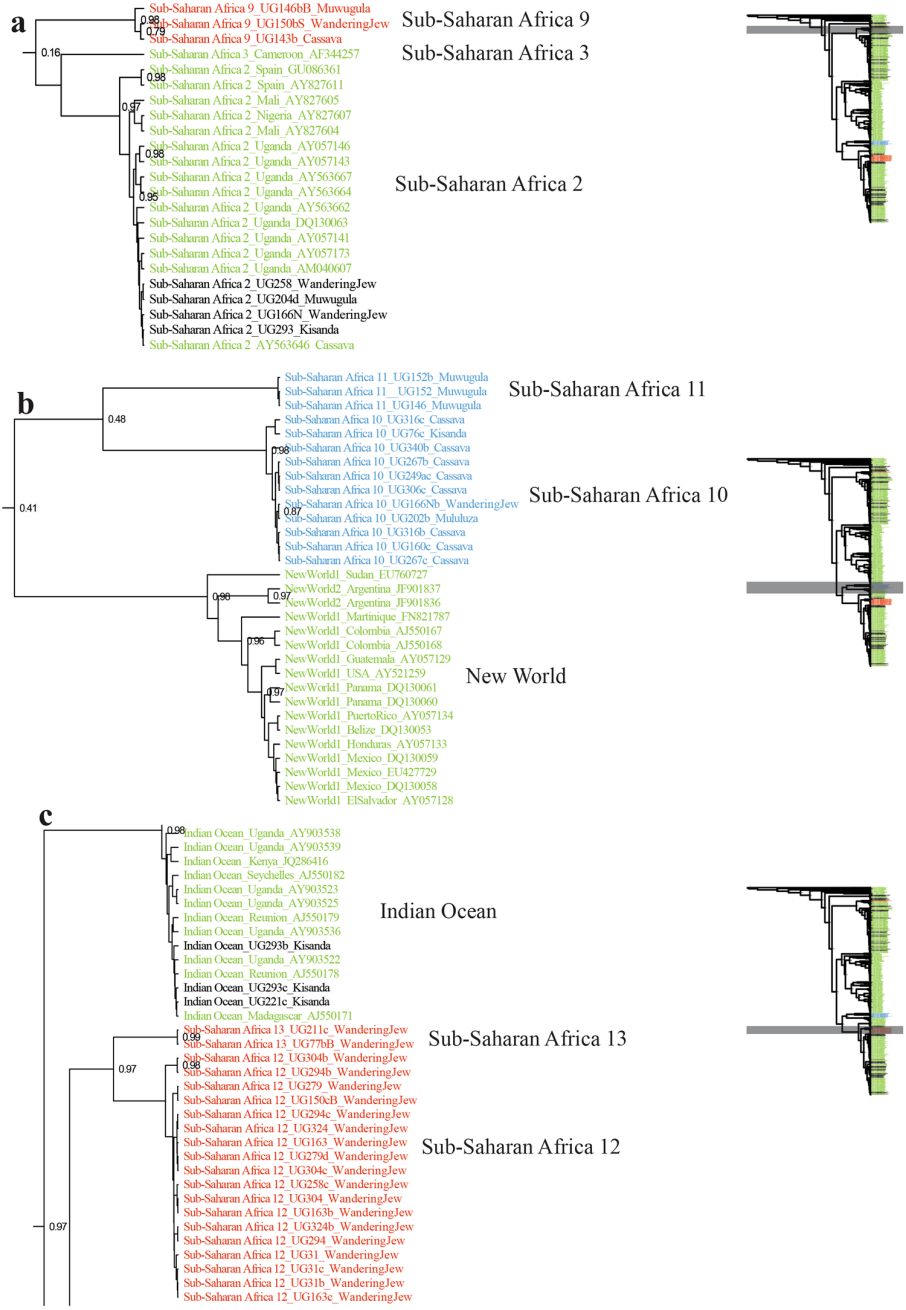
The newly identified putative species are shown in the MrBayes tree. Three sections (**a**–**c**) of the entire phylogenetic tree that are highlighted in grey are expanded in the sub-figures adjacent to them. The new putative species are highlighted in red (Sub-Saharan Africa 9, Sub-Saharan Africa 12, and Sub-Saharan Africa 13) and blue text (Sub-Saharan Africa 10 and Sub-Saharan Africa 11), respectively. The newly discovered link between African and New World *B. tabaci* are shown in sub-figure b. Reference sequences from GenBank used in the analysis appear in green text. Previously reported putative species also found during this study are highlighted in black text.

Based on the criterion that sequence divergence of more than 3.5‒4.0% indicates different *B. tabaci* species^2,45^, twelve *B. tabaci* species and one non-*tabaci* species were identified from the partial 121 *mtCO1* sequences. Among them, five new previously undescribed putative species are reported for the first time here, which have been named Sub-Saharan Africa 9 to 13 (SSA9 – SSA13). Two of these putative new species, Sub-Saharan Africa 10 (SSA10) and Sub-Saharan Africa 11 (SSA11) clustered away from the other African species and next to species named New World 1 and New World 2 from the Americas (Fig. 4b), with which they shared 84.8‒86.5% sequence identity (Table 2). This provides new evidence for a close evolutionary link between African and American *B. tabaci* species, with the SSA10‒11 clade most closely related to the New World clade (Supplementary Fig. S1).

**Table 2:** Percentage nucleotide identity of Ugandan whitefly sequences (from *B. tabaci* and non-*tabaci* species) to their closest relatives in GenBank.

*Bemisia tabaci* and non-*tabaci* species	Number of sequences	Closest relative GenBank accession no.	% sequence identity with closest relative
SSA1	19	AY903463	98.4‒100%
SSA2	4	AY057173	99.6%
SSA6	5	AY903561	99.7‒99.9%
**SSA9**	3	AY903463	92.8‒93.5%
**SSA10**	11	FN821787	85.3‒86.0%
**SSA11**	3	FN821787	84.8%
**SSA12**	18	JX993192	93.9‒94.1%
**SSA13**	2	EU760753	94.6‒94.7
MEAM1	1	KC661294	99.9%
MEAM2	7	AJ550177	97.1%
MED	32	EU760732	96.5‒99.9%
Indian Ocean	3	AY903538	99.1‒99.7%
*B*. Uganda1	13	AY903575	99.6‒100%

The new putative species identified in this study appear in bold text. The abbreviations for the whitefly species are as follows: SSA is sub-Saharan Africa, MEAM is Middle East-Asia Minor, MED is Mediterranean and *B*. Uganda 1 is *Bemisia* Uganda 1.

The global phylogeny of the 121 sequences, analysed with 570 reference sequences for known global *B. tabaci* species is shown in the Supplementary Figure S1. The topology showed that the *B*. Uganda1 species, together with *B. atriplex* and *B*. Japan2 were basal to the monophyletic group of *B. tabaci* species (Supplementary Fig. S1). *B*. Uganda1 was present on the five weed species, but not on cassava, confirming previous reports^12,46,47^ (Table 1) that cassava is not one of the hosts of this whitefly species. The Sub-Saharan Africa clade was basal to all other clades within the *B. tabaci* group of species (Supplementary Fig. S1) and was composed of SSA1, SSA2, SSA3, SSA4, SSA5, SSA6, SSA8 and SSA9 species. In our sample collection, SSA1, SSA2, SSA6 and SSA9 were identified.

In the previous literature, there have been two reports designating SSA5 to new putative *B. tabaci* species identified on cassava in Uganda and South Africa^20,36^. Our current study opted to rename the SSA5 (accession number: AM040598), identified by Boykin *et al*.^36^ on cassava in Uganda, as SSA8, while retaining the name SSA5 for the *B*. *tabaci* species identified on cassava and other weeds in South Africa by Esterhuizen *et al*.^20^ (accession number: JN104719). The species collected on wild mint previously called “Uganda 3”^12^ (accession number: AY903561) was named SSA6 here for consistency following the new naming system for *B. tabaci* species.

New *mtCO1* sequences were obtained for previously recognised species in the SSA clade (SSA1, SSA2, SSA6), and these shared 98.4‒100% sequence identity with their closest relatives (AY903463, AY057173 and AY903561) in GenBank (Table 2). We identified SSA9, however, as a new species that clustered with SSA1–SSA6 in what has previously been referred to as the SSA clade^2,48^ (Fig. 4a) and it shared 92.8‒93.5% sequence identity with its closest relative, UgCsNm3 (AY903463) in GenBank (Table 2).

The Italy and Australia-Asia clades were basal to the New World and Africa-Middle East Asia Minor clades (Supplementary Fig. S1), the former being composed of Italy1, Morocco, SSA7 (from Cameroon), Japan 1, Chinese, Asian and Australian species, none of which were found in Uganda. Fourteen *mtCO1* sequences obtained in this study clustered with sequences assigned previously to the New World clade. The New World clade was basal to the Africa-Middle East-Asia Minor clade (Supplementary Fig. S1), the latter being composed of IO, MED, MEAM1, MEAM2, SSA12 and SSA13 species, which were all found in Uganda. The IO, MED, MEAM1, MEAM2 sequences obtained from our samples shared 96.5–99.9% sequence identity with their closest relatives in GenBank (Table 2). The putative new species SSA12 and SSA13, however, shared only 93.9–94.7% sequence identity with their closest relatives in GenBank (Table 2). SSA12 and SSA13 grouped next to each other and were in the Africa-Middle East-Asia Minor clade (Fig. 4c).

### **Estimated global divergence of*B. tabaci*species**

To test whether or not the discovery of the new species altered our view of the evolutionary history of *B. tabaci*, the global partial *mtCO1* data-set was re-analysed together with the sequences generated in this study. We considered that the discovery of SSA10 and SSA11 and their close link to the New World species could provide a good test of the hypothesis that the separation of South America from Africa, which occurred between 120 to 84 mya^38^, was responsible for this evolutionary split. For this hypothesis to be correct, we would predict that divergence of Old World from the New World *B*. *tabaci* species should occur over a similar time-frame.

**Figure 5:**
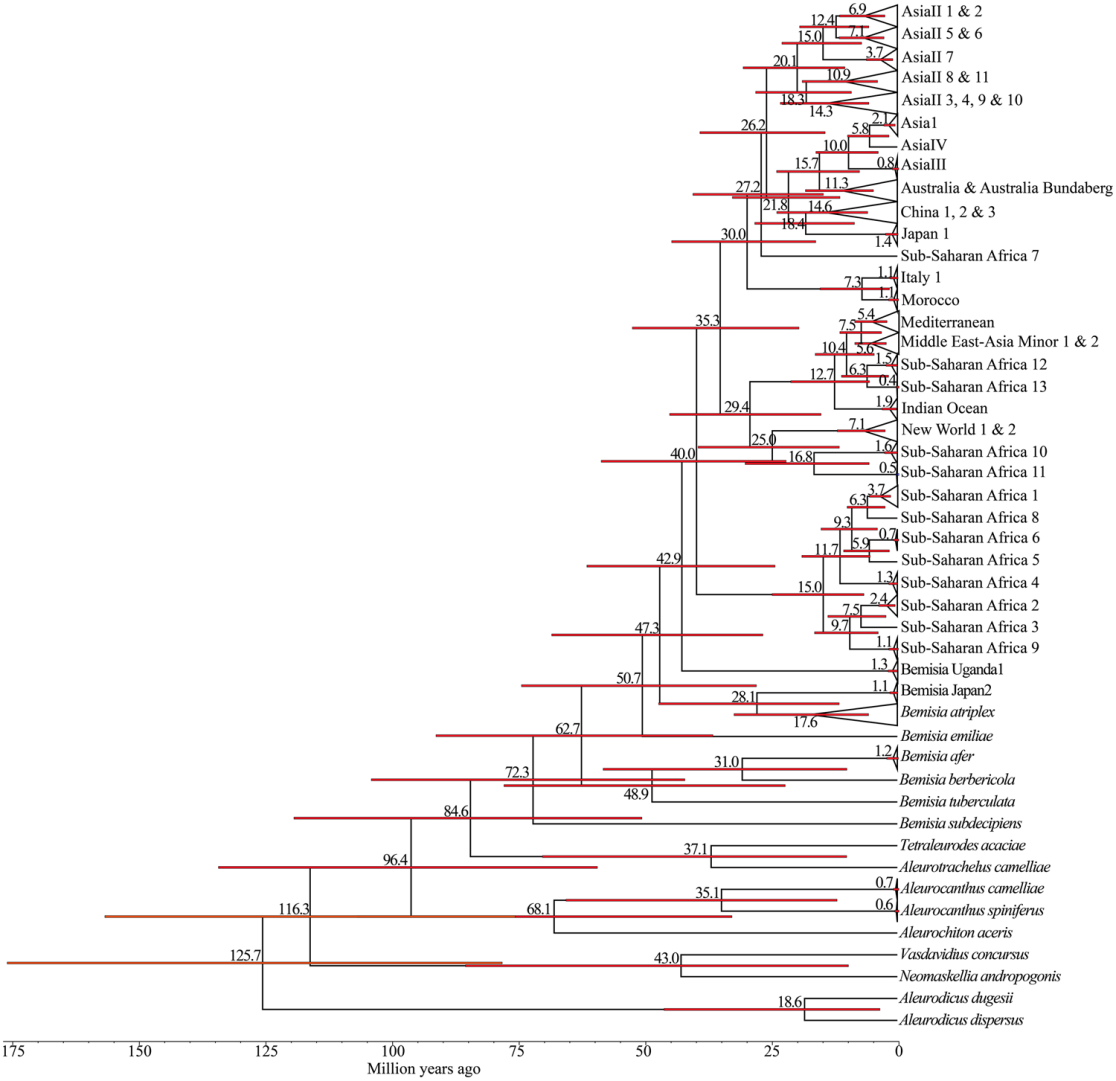
Time-calibrated phylogenetic tree of *B. tabaci* based on partial *mtCO1* sequences. Divergence estimates expressed in million years ago (mya) are shown above the branches with 95% confidence intervals (red bars).

Our molecular clock analysis showed that *B. tabaci* diverged from the rest of *Bemisia* species *c*. 40 mya. (Fig. 5), where it split into two major groups: (i) the SSA clade and (ii) Africa-Middle East-Asia Minor, New World, Italy and Australia-Asia clades (Fig. 5). Basal to the split of *B. tabaci* species was the SSA clade whose members split into two groups about 15 mya. The first group composed of the SSA2, SSA3 and SSA9 species split *c*. 10 mya and diversified *c*. 8‒1 mya, while the second group was composed of the SSA1, SSA4, SSA5, SSA6, and SSA8 species split *c*. 12 mya and diversification occurred 6–1 mya.

The Africa-Middle East-Asia Minor clade (composed of IO, MED, MEAM1, MEAM2, SSA12 and SSA13) split from the New World clade (composed of New World 1, New World 2, SSA10 and SSA11 species) *c*. 29 mya. About 25 mya, the SSA10 and SSA11 species split from the New World species and diversified about 2‒1 mya. Diversification within the New World species occurred *c*. 7–1 mya. About 13 mya, the IO species split from MED, MEAM1/MEAM2, SSA12 and SSA13 species. Diversification within the IO species occurred *c*. 2 mya. The SSA12 and SSA13 species split from MED, MEAM1 and MEAM2 species *c*. 10 mya. Diversification within SSA12 and SSA13 species occurred 2 mya and for the MED, MEAM1 and MEAM2 species, *c*. 6–5 mya.

Approximately 35 mya, the Italy and Australia-Asia clade split from the Africa-Middle East-Asia Minor and New World clades. The Italy clade (Italy 1 and Morocco) then diverged from the Australia-Asia clade (SSA7, Japan1, China, Australia and Asian species) about 30 mya and diversified about 1 mya. Within the Australia-Asia clade, the SSA7 split from Japan1, China, Australia and Asian species at *c*. 27 mya. The AsiaII split from Australia, Asia1, AsiaIII, Asia IV, China and Japan 1 species *c*. 26 mya and the former species diversified 18–1 mya. Diversification within the AsiaII occurred 15–4 mya.

## Discussion

This study is the first to report *mtCO1* sequences from *B. tabaci* species in the Old World (Africa) clustering close to the New World species and thus provides the first strong evidence for a close evolutionary link between the Old (African) and New World species. The development of a new primer set for amplification of the *mtCO1* ‘barcode’ of *B. tabaci* species enabled 12 putative *B. tabaci* species to be identified from the Ugandan whitefly samples, of which five were new including the two (SSA10 and SSA11) most closely related to the New World species (Fig. 4).

Gueguen *et al*.^22^ reported a single specimen from the Sudan (EU760727) that was apparently a New World species (according to the 3.5% *mtCO1* divergence criterion), most probably a recent introduction from the New World into Africa by human trade. The *mtCO1* sequence of the Sudanese sample is quite different from the SSA10 and SSA11 sequences that were amplified from samples collected from multiple locations in our study (Table 1).

In addition to the SSA10 and SSA11 species, 10 other putative species of *B. tabaci* and a non-*tabaci* species were identified amongst the whitefly samples collected from weeds. Of the 10, three were new putative species and were named: SSA9, SSA12 and SSA13. The three new putative species, SSA9 and SSA12 and SSA13, grouped with the SSA and Africa-Middle East-Asia Minor higher-level genetic groups (11% level or greater *mtCO1* sequence divergence), respectively, identified by Dinsdale *et al*.^2^. The rest of the seven species (IO, MED, MEAM1, MEAM2, SSA1, SSA2 and SSA6) were reported previously in Uganda^12,19,21^ and elsewhere^49^. This high genetic diversity present in the study area further supports SSA and equatorial Africa, in particular, as the centre of origin of *B. tabaci*^26,50^.

MEAM2 *B*. *tabaci* species identified in this study shared 97.1% sequence similarity with the closest published partial *mtCO1* DNA of a whitefly individual from Reunion (AJ550177). Similar to our study, previous studies carried out in Reunion^51^, Japan^49^ and Turkey^52^ identified this whitefly as a distinct species within the *B*. *tabaci* species complex. However, analysis of mitogenomes of three Peruvian individuals expected to be MEAM2 based on the Sanger sequence derived partial *mtCO1* DNA revealed that they were MEAM1^53^. The misidentification of MEAM2 sequences based on the partial *mtCO1* DNA was attributed to the amplification of nuclear mitochondrial DNA (NUMTS) or PCR artefacts such as DNA polymerase-introduced errors. Although partial *mtCO1* sequences of MEAM2 species are reported in this study, a full mitogenome of this species has been generated from a single whitefly collected in Uganda. This confirms that the MEAM2 species generated in this study and currently occurring in Uganda is genuine and was not as result of NUMTS or PCR artefacts as reported by Tay *et al*.^53^.

*Bemisia* Uganda1 was also found by our study to occur in Uganda and this has been previously included in the *B. tabaci* group^12,54^. Our new analysis, however, shows that *B*. Uganda1 and *Bemisia* Japan2 (AB308110)^7^ clearly group outside the *B. tabaci* complex (Supplementary Fig. S1), although the adults of the former are morphologically indistinguishable (H. Mugerwa, personal observation). The *mtCO1* marker provides only limited phylogenetic information and so further genetic information, such as that provided by single-copy nuclear genes, is required to conclude with more certainty whether or not *Bemisia* Uganda1 is a member of the *B. tabaci* species complex.

The estimated date of divergence of SSA10 and SSA11 species from the New World clade was *c*. 25 mya (Fig. 5) and the separation of South America from Africa occurred over a prolonged period of 120–84 mya^38^. Our new data and analyses, therefore are clearly inconsistent with the hypothesis that divergence of the New and Old World species corresponded to the geological separation of South America from Africa. In addition, New World 1 is ancestral to New World 2 and this could be explained by the invasion of New World 1 into South America when the two land masses became joined *c*. 3 mya^55,56^. This hypothesis can only be tested more rigorously when, and if, more whitefly fossils, molecular data and further key species that cluster within the *B. tabaci* group are discovered.

As an alternative hypothesis, our estimated date of divergence of SSA10 and SSA11 species from the New World species corresponds with the most plausible date for the Old World/New World split in begomoviruses, which was estimated to be *c*. 20‒30 mya^50^. The presence of the Beringian land bridge connecting Asia and Western North America and a warm temperate climate between *c*. 65‒35 mya, enabled considerable exchange of terrestrial fauna and flora^57^ between these land masses. Movement of early whitefly-transmitted begomoviruses between Asia and North America via the Beringian land bridge would therefore have been possible up to *c*. 35 mya^50^. Our new data and the intimate relationship between begomoviruses and their *B. tabaci* vector species, therefore, strongly support the hypothesis that invasion of the New World occurred into North America through the Beringian land bridge and subsequently into Latin America. In addition, the SSA and Africa-Middle East-Asia Minor clade diverged from Italy and Australia-Asia clades between *c*. 35 mya. The warm temperate climate across the globe during that period would have enabled further movement and speciation amongst the *B*. *tabaci* major clades.

Our new estimates of when the members of the *B. tabaci* species diverged also differed from those of other researchers. Santos-Garcia *et al*.^41^, for example, reported that diversification within MEAM1 and MED species took place 0.63–0.16 mya (estimated using runAB and BEAST2) and 2.88–0.44 mya (estimated using PhyloBayes3). Boykin *et al*.^36^ estimated diversification of MEAM1 and MED species at about 13 mya. We estimate the diversification of MEAM1 and MED occurred *c*. 6–5 mya. Different outgroup calibration dates ranging from 263‒125 mya and full (1,341 bp) *vs* partial (657 bp) *mtCO1* gene sequences were used in these different analyses. We conclude that more precise divergence estimates for *B*. *tabaci* species are only likely to be attained when additional fossil specimens within *Bemisia* are discovered and used to set accurate calibration points in geological time. We also consider that nuclear markers shall also be required, because they may reveal different evolutionary histories to the mitochondrial genome^58^.

Sub-Saharan Africa 7 that links Asia to Africa^22^, the Morocco species that links Italy to Africa^37^, as well as SSA10 and SSA11 that link the New World to Africa, all provide further evidence that members of the *B. tabaci* complex have a common ancestor that originated in Sub-Saharan Africa. Since SSA7 is represented by only one sequence in GenBank, we suggest that further collections be made in the region where SSA7 was found, to strengthen the weight of evidence for this theory.

The majority of studies on East African cassava whiteflies in the previous decade have focussed mainly on cassava and the two known *B. tabaci* species, SSA1 and SSA2 that colonise it^1,12,19,21,35^. The new species reported here were not found in these earlier studies and this is particularly surprising for SSA10, because its preferred host-plant appears to be cassava (Fig. 3b) and it was detected in six districts in our study. The most probable reason for this is that the primer set used by whitefly researchers globally has generally been the MT10/C1-J-2195/MT12/TL2-N-3014 pair. Figure 1 shows that there are six annealing mismatches in the MT12/TL2-N-3014 primer and the *B. tabaci* mitogenome sequences obtained by independent means^59–62^. Given the clear problems with the MT10/C1-J-2195/MT12/TL2-N-3014 primer set, it is surprising that they have been used so widely in the past. The modifications described here, however, will help reveal the true complexity and diversity within the *B. tabaci* groups of species.

The 2195Bt and C012/Bt-sh2 primer set designed in this study produced PCR amplicons of the same size as the MT10/C1-J-2195 and MT12/TL2-N-3014 primer set because both primer sets were designed from the same positions (Fig. 1). In contrast to Shatters *et al*.^30^ study, a modified primer set designed efficiently amplified partial *mtCO1* gene for *Bemisia* and some related Aleyrodidae, however, it produced relatively short PCR amplicons (~748 bp) compared to the MT10/C1-J-2195 and MT12/TL2-N-3014 primer set. The relatively short PCR amplicon was due to Shatters *et al*.^30^ forward primer (Btab-uni-PrimerR) designed 52 base pairs downstream from the MT10/C1-J-2195 forward primer. In an era when more than 99% of species that ever lived on earth are now extinct^63,64^, our study reports the discovery of five new species within the *B. tabaci* species complex and suggests that with more sampling in Sub-Saharan Africa and the use of more efficient primers, it is probable that many more species will be discovered. In addition to the partial *mtCO1* marker used to identify new putative species in this study, we recommend that biological studies such as mating crosses be carried out between the new identified putative species and other SSA species. These findings (new putative species) also require us to re-evaluate current models and the time-frame of whitefly evolution. As low-cost genome sequencing becomes increasingly available, the proposed model for evolution should be expanded and validated by including a range of phylogenetically informative nuclear genes. Comparative genomic studies will then become a resource to catalyse the development of novel tools and technologies to manage these economically devastating pest species.

## Materials and Methods

### Whitefly field collections

Adult whiteflies were collected from cassava (*Manihot esculenta* Crantz) (n = 9) and five weed species: Wandering Jew (*Commelina benghalensis* L.) (n = 37), Muwugula (*Pavonia urens* Cav.) (n = 27), Mexican fire plant (*Euphorbia heterophylla* L.) (n = 16), Joobyo (*Cleome gynandra* L.) (n = 14), Mululuza (*Vernonia amygdalina* Delie) (n = 18), which occur frequently within and/or the surroundings of cassava fields across 26 districts in Uganda between July and August 2013 (Table 1 and Supplementary Dataset). Collections were made using an aspirator and the whitefly samples stored in 1.5-ml Eppendorf tubes containing 90% ethanol. Samples from the same host plant species at each site were stored in the same Eppendorf tube. For each collection site, the geo-coordinates (latitude and longitude) were recorded using a Geographical Positioning System (GPS, Garmin eTrex Vista Cx) together with the village and district name. The samples were maintained at room temperature during field collection in Uganda, but stored at −20 °C upon arrival at Natural Resources Institute (NRI), UK until molecular analysis.

### Generation of a map

The geo-coordinates for each collection site were used to generate a map (Fig. 3) using the ArcMap version 10.2.2 software program (http://www.arcgis.com/features/).

### **Global*B. tabaci*samples and outgroups**

The data set of *B. tabaci* and outgroup sequences representing global diversity was obtained from GenBank. Five hundred seventy individual 657 bp *mtCO1* sequences’ 3′-end region were selected and used in the analysis. Published host data were obtained for each sample. Outgroup taxa were selected based on their morphological similarities to the fossil *Aleurodicus burmiticus*^43,65^. Of the selected 570 sequences, 31 individual outgroup sequences representing 19 other whitefly species were used in the analysis. Uganda and Japan2 species grouped most closely to *B. emiliae* and *B. atriplex*. To avoid the confusion caused by their unusual names, we henceforth refer to Uganda and Japan2 species as, *Bemisia* Uganda1 and *Bemisia* Japan2, respectively.

### Whitefly DNA extraction

Three adult whiteflies were selected randomly from those collect for each sampled plant species and location. Genomic DNA was extracted from single whiteflies by crushing each in 50 µl of 10% (w/v) Chelex 100 sodium form solution (Sigma Aldrich, St Louis, USA) in a 1.5-ml Eppendorf tube using a sterile plastic rod^66^. The extracts were incubated for 20 min at 56 °C and then for 5 min at 100 °C. Subsequently, extracts were centrifuged for 5 min at 15,900 *g* in a 5424 R Eppendorf centrifuge (Eppendorf UK Limited) and placed immediately on ice prior to proceeding to using the individual whitefly extracts as templates for PCR amplification.

### Mitochondrial DNA amplification and sequencing

Amplification of a partial fragment (867 bp) of the *mtCO1* gene was performed using the forward primer MT10/C1-J-2195 (5′-TTGATTTTTTGGTCATCCAGAAGT-3′) in combination with a reverse primer MT12/TL2-N-3014 (5′-TCCAATGCACTAATCTGCCATATTA-3′)^29^. The PCR reaction mixture (20 µl) contained 10 µl of 2× reSource™ Taq Mix (Source BioSciences, UK), 1 µl of each primer stock (10 µM), 6 µl of molecular biology grade water (Sigma Aldrich, St Louis, USA) and 2 µl of DNA template. Initial denaturation of template DNA was conducted at 94 °C for 2 min followed by 35 cycles of denaturation at 94 °C for 20 s, primer annealing at 52 °C for 30 s and extension at 72 °C for 1 min. The final extension of 10 min was run at 72 °C and the reaction held at 4 °C in a 2720 Applied Biosystems thermal cycler (Applied Biosystems, UK). Electrophoresis of PCR products was performed on 2% (w/v) agarose gels in 0.5× TBE buffer stained using RedSafe™ according to manufacturer’s guidelines (iNtRON BIOTECHNOLOGY, Korea). PCR products were visualised under UV light (302 nm) and those of the expected (~867 bp) size were purified for sequencing and DNA cloning using a reSource™ PCR purification multipack kit (Source BioScience, UK) as per the manufacturer’s procedure. Purified PCR products were cloned in three replicates using the pGEM®-T easy vector kit (Promega, UK) as per the manufacturer’s instructions. Purified and cloned PCR-amplified products were sent for sequencing at Source BioScience, UK.

### **Modified*mtCO1*primers**

Due to mismatches observed in the MT10/C1-J-2195 and MT12/TL2-N-3014 primer pair to *B. tabaci* mitogenome sequences (Asia I: KJ778614, MED: JQ906700, New World I: AY521259 and *B*. *afer*: KF734668), a modified primer set that most matches the *B. tabaci* sequences was designed (Fig. 1). This was done through multiple sequence alignment of *mtCO1* gene sequences (nucleotide positions: ~600 to 1700) available in GenBank for complete mitogenomes and unpublished sequences of various species of the *B. tabaci* complex (Asia I, New World, MED and SSA1) and *B. afer* using MAFFT v7.273^67^. The alignment block was edited and illustrated using TEXshade program^68^. Regions where the MT10/C1-J-2195 and MT12/TL2-N-3014 primer pair aligned were identified (Fig. 1). The modified primer set: 2195Bt (5′-TGRTTTTTTGGTCATCCRGAAGT-3′) and C012/Bt-sh2 (5′-TTTACTGCACTTTCTGCC-3′) was designed to anneal to the same region as the MT10/C1-J-2195 and MT12/TL2-N-3014 primer pair to ensure compatibility of sequences for phylogenetic analyses (Fig. 1). The PCR reaction mixture and conditions of the new primer set were the same as for the MT10 & MT12 primer set.

### Phylogenetic analysis

Whitefly *mtCO1* sequences were aligned together with reference whitefly sequences obtained from GenBank in Geneious v 9.1 with the MUSCLE^69^ alignment option set to 50 iterations, then visually inspected and manually adjusted where necessary. The final alignment was translated to ensure sequences were aligned within the correct reading frame.

The model of molecular evolution was determined using Modeltest 3.6^70^. MrBayes 3.2.2^71^ was used to partition data based on codon position in the alignment. Codon positions 1 and 2 were treated with the following commands in MrBayes: lsetapplyto = (1,2) nst = 2 rates = gamma and the third position: lsetapplyto = (3) nst = 6 rates = gamma. MrBayes 3.1.2 was run for 50 million generations and trees were sampled every 1000 generations. All runs reached a plateau in likelihood score (i.e. stationarity), which was indicated by the standard deviation of split frequencies (0.0015), and the potential scale reduction factor (PSRF) was close to one, indicating the MCMC chains converged. Convergence of the runs was also checked using Tracer v1.6 and the effective sample size (ESS) values were well above 200 for each run. Twelve thousand five hundred trees were suboptimal at the beginning of the runs and were therefore discarded.

### Divergence estimates

BEAUti v1.8.2^72^ was used to generate the xml file for the multiple BEAST runs. Four independent runs of BEAST were conducted following the model sets Boykin *et al*.^36^. The tree height prior was set to a normal distribution, with an initial value of 123, to represent the age of the fossil 130 mya^43^ with a standard deviation of 20. The MCMC were run for 50 million generations and sampled every 1000th generation. Convergence of the multiple runs was checked using Tracer v1.6.0 and the ESS values were well above 200 for each run. Two independent BEAST runs were completed and the two tree files were combined using Logcombiner. The trimmed output was combined to yield posterior estimates of mean.rate, coefficient variation in rates, ucld.mena, ucld.stdev and the mean and 95% highest posterior density. TreeAnnotator was used to generate a final tree, which was viewed in FigTree v1.4.2.

## Electronic supplementary material

Supplementary Information

Supplementary Dataset 1
