# Supplementary material for: African ancestry of New World, Bemisia tabaci-whitefly species
Source: Sci Rep. 2018 Feb 9;8:2734. doi: 10.1038/s41598-018-20956-3 (PMC5807539; doi:10.1038/s41598-018-20956-3)
Supplement: Supplementary file 1 — Supplementary Information [file 41598_2018_20956_MOESM1_ESM.pdf]

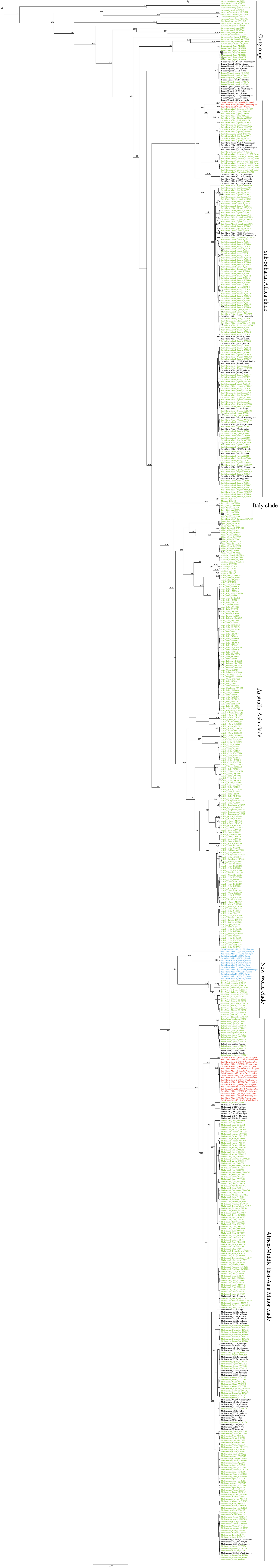

Outgroups
Sub-Saharan Africa clade
Italy clade
Australia-Asia clade
New World clade
Africa-Middle East-Asia Minor clade

Supplementary Figure 1: MrBayes phylogeny for *Bemisia tabaci* generated using 30 million generations, trees sampled every 1000 generations. The new putative species are highlighted in red and blue. The newly discovered link between African *B. tabaci* (Sub-Saharan Africa 10 and Sub-Saharan Africa 11) and New World are highlighted in blue text. The 570 reference sequences used in the analysis are highlighted in green. The previously reported putative species identified during this study are highlighted in black.

Title Page

African ancestry of New World, *Bemisia tabaci*-whitefly species

Habibu Mugerwa[1,2], Susan Seal[1], Hua-Ling Wang[1], Mitulkumar V. Patel[1], Richard Kabaalu[2], Christopher A. Omongo[2], Titus Alicai[2], Fred Tairo[3], Joseph Ndunguru[3], Peter Sseruwagi[1, 3] & John Colvin[1*]

[1]Natural Resources Institute, University of Greenwich, Central Avenue, Chatham Maritime, Kent, ME4 4TB, UK.
[2]Root Crops Programme, National Crops Resources Research Institute, P. O. Box 7084, Kampala, Uganda.
[3]Biotechnology Department, Mikocheni Agricultural Research Institute, P.O. Box 6226, Dar es Salaam, Tanzania.

Correspondence and request of material should be addressed to J. C. (email: j.colvin@greenwich.ac.uk).
