# Supplementary material for: African ancestry of New World, Bemisia tabaci-whitefly species
Source: Sci Rep. 2018 Feb 9;8:2734. doi: 10.1038/s41598-018-20956-3 (PMC5807539; doi:10.1038/s41598-018-20956-3)
Supplement: Supplementary file 2 — Supplementary Dataset 1 [file 41598_2018_20956_MOESM2_ESM.docx]

**Title Page**

**African ancestry of New World, *Bemisia tabaci*-whitefly species**

Habibu Mugerwa^1,2^, Susan Seal^1^, Hua-Ling Wang^1^, Mitulkumar V. Patel^1^, Richard Kabaalu^2^, Christopher A. Omongo^2^, Titus Alicai^2^, Fred Tairo^3^, Joseph Ndunguru^3^, Peter Sseruwagi^1, 3^ & John Colvin^1*^

^1^Natural Resources Institute, University of Greenwich, Central Avenue, Chatham Maritime, Kent, ME4 4TB, UK.

^2^Root Crops Programme, National Crops Resources Research Institute, P. O. Box 7084, Kampala, Uganda.

^3^Biotechnology Department, Mikocheni Agricultural Research Institute, P.O. Box 6226, Dar es Salaam, Tanzania.

Correspondence and request of material should be addressed to J. C. (email: j.colvin@greenwich.ac.uk).

| **District** | **Village** | **Sample no.** | **Host plant** | **Whitefly species** | **Accession no.** | **Longitude** | **Latitude** |
| --- | --- | --- | --- | --- | --- | --- | --- |
| Nakasongola | Kiimatwe | UG221c | Kisanda | Indian Ocean | KX570749 | 32.07686 | 1.30038 |
| Ngora | Akeit | UG293b | Kisanda | Indian Ocean | KX570750 | 33.49901 | 1.35491 |
| Ngora | Akeit | UG293c | Kisanda | Indian Ocean | KX570751 | 33.49901 | 1.35491 |
| Budaka | Kamonkoli | UG315 | Muwugula | Mediterranean | KX570752 | 34.0589 | 1.04752 |
| Budaka | Kamonkoli | UG315b | Muwugula | Mediterranean | KX570753 | 34.0589 | 1.04752 |
| Bukomansimbi | Kyabiri | UG50b | Joobyo | Mediterranean | KX570754 | 31.60107 | -0.26234 |
| Bukomansimbi | Kyabiri | UG50c | Joobyo | Mediterranean | KX570755 | 31.60107 | -0.26234 |
| Hoima | Kinogozi | UG159bB | Muwugula | Mediterranean | KX570756 | 31.40287 | 1.36103 |
| Hoima | Kinogozi | UG159 | Muwugula | Mediterranean | KX570757 | 31.40287 | 1.36103 |
| Kaberamaido | Ogasia | UG279c | Wandering Jew | Mediterranean | KX570758 | 33.13734 | 1.53665 |
| Kayunga | Ntooke | UG352c | Mululuza | Mediterranean | KX570759 | 32.53753 | 0.41086 |
| Kibaale | Kilanzi | UG152c | Muwugula | Mediterranean | KX570760 | 30.93841 | 1.07421 |
| Kiboga | Bukomero | UG174b | Muwugula | Mediterranean | KX570761 | 32.04367 | 0.69042 |
| Kiboga | Sinde | UG170bB | Joobyo | Mediterranean | KX570762 | 31.94805 | 0.78855 |
| Kyenjojo | Mukole | UG146c | Muwugula | Mediterranean | KX570763 | 30.72763 | 0.76229 |
| Lira | Awangdyiang | UG266 | Muwugula | Mediterranean | KX570764 | 32.55022 | 2.10065 |
| Luweero | Butanze | UG204b | Muwugula | Mediterranean | KX570765 | 32.30955 | 0.44537 |
| Luweero | Butanze | UG204c | Muwugula | Mediterranean | KX570766 | 32.30955 | 0.44537 |
| Mpigi | Mazimbamutubagumu | UG71c | Joobyo | Mediterranean | KX570767 | 32.34638 | 0.21412 |
| Oyam | Ayalo | UG254 | Muwugula | Mediterranean | KX570768 | 32.3306 | 2.15646 |
| Oyam | Ayalo | UG254b | Muwugula | Mediterranean | KX570769 | 32.3306 | 2.15646 |
| Rakai | Rusolo | UG9 | Joobyo | Mediterranean | KX570770 | 31.48055 | -0.69964 |
| Rakai | Rusolo | UG9b | Joobyo | Mediterranean | KX570771 | 31.48055 | -0.69964 |
| Rakai | Rusolo | UG9c | Joobyo | Mediterranean | KX570772 | 31.48055 | -0.69964 |
| Wakiso | Busami | UG83 | Wandering Jew | Mediterranean | KX570773 | 32.39158 | 0.16002 |
| Wakiso | Busami | UG83bB | Wandering Jew | Mediterranean | KX570774 | 32.39158 | 0.16002 |
| Wakiso | Busami | UG83cB | Wandering Jew | Mediterranean | KX570775 | 32.39158 | 0.16002 |
| Wakiso | Butaku | UG178b | Joobyo | Mediterranean | KX570776 | 32.28541 | 0.51368 |
| Luweero | Butanze | UG21 | Mululuza | Middle East Asia Minor 1 | KX570777 | 31.66463 | -0.44107 |
| Wakiso | Butaku | UG315c | Mululuza | Middle East Asia Minor 2 | KX570778 | 34.0589 | 1.04752 |
| Wakiso | Butaku | UG159c | Mululuza | Middle East Asia Minor 2 | KX570779 | 31.40287 | 1.36103 |
| Wakiso | Butaku | UG174 | Mululuza | Middle East Asia Minor 2 | KX570780 | 32.04367 | 0.69042 |
| Wakiso | Butaku | UG174c | Mululuza | Middle East Asia Minor 2 | KX570781 | 32.04367 | 0.69042 |
| Mpigi | Mazimbamutubagumu | UG220 | Joobyo | Middle East Asia Minor 2 | KX570782 | 32.07686 | 1.30038 |
| Wakiso | Butaku | UG220b | Mululuza | Middle East Asia Minor 2 | KX570783 | 32.07686 | 1.30038 |
| Masaka | Bukona | UG220c | Muwugula | Middle East Asia Minor 2 | KX570784 | 32.07686 | 1.30038 |
| Budaka | Nakatende | UG35 | Muwugula | SubSaharan Africa 1 | KX570785 | 31.69656 | -0.28751 |
| Hoima | Kinogozi | UG35b | Muwugula | SubSaharan Africa 1 | KX570786 | 31.69656 | -0.28751 |
| Kiboga | Bukomero | UG35c | Muwugula | SubSaharan Africa 1 | KX570787 | 31.69656 | -0.28751 |

| **District** | **Village** | **Sample no.** | **Host plant** | **Whitefly species** | **Accession no.** | **Longitude** | **Latitude** |
| --- | --- | --- | --- | --- | --- | --- | --- |
| Kiboga | Bukomero | UG50 | Muwugula | SubSaharan Africa 1 | KX570788 | 31.60107 | -0.26234 |
| Nakasongola | Kiimatwe | UG193b | Mululuza | SubSaharan Africa 1 | KX570789 | 32.32081 | 0.33633 |
| Nakasongola | Kiimatwe | UG77 | Mululuza | SubSaharan Africa 1 | KX570790 | 32.34638 | 0.21412 |
| Nakasongola | Kiimatwe | UG77c | Mululuza | SubSaharan Africa 1 | KX570791 | 32.34638 | 0.21412 |
| Mpigi | Mazibamutubagumu | UG71b | Wandering Jew | SubSaharan Africa 1 | KX570792 | 32.34638 | 0.21412 |
| Mpigi | Muduuma | UG76 | Wandering Jew | SubSaharan Africa 1 | KX570793 | 32.34638 | 0.21412 |
| Mpigi | Muduuma | UG76b | Wandering Jew | SubSaharan Africa 1 | KX570794 | 32.34638 | 0.21412 |
| Mpigi | Mazibamutubagumu | UG92 | Wandering Jew | SubSaharan Africa 1 | KX570795 | 32.22419 | 0.34878 |
| Mpigi | Muduuma | UG92b | Wandering Jew | SubSaharan Africa 1 | KX570796 | 32.22419 | 0.34878 |
| Oyam | Ayalolor | UG92cS | Muwugula | SubSaharan Africa 1 | KX570797 | 32.22419 | 0.34878 |
| Bukomansimbi | Budda | UG221 | Kisanda | SubSaharan Africa 1 | KX570798 | 32.07686 | 1.30038 |
| Bukomansimbi | Budda | UG221b | Kisanda | SubSaharan Africa 1 | KX570799 | 32.07686 | 1.30038 |
| Bukomansimbi | Budda | UG254c | Kisanda | SubSaharan Africa 1 | KX570800 | 32.3306 | 2.15646 |
| Bukomansimbi | Kyabiri | UG86 | Joobyo | SubSaharan Africa 1 | KX570801 | 32.43004 | 0.17345 |
| Luweero | Bajjomituba | UG86bB | Kisanda | SubSaharan Africa 1 | KX570802 | 32.43004 | 0.17345 |
| Mpigi | Mazimbamutubagumu | UG86cB | Joobyo | SubSaharan Africa 1 | KX570803 | 32.43004 | 0.17345 |
| Mpigi | Mazimbamutubagumu | UG316b | Kisanda | SubSaharan Africa 10 | KX570804 | 34.0589 | 1.04752 |
| Mpigi | Mazimbamutubagumu | UG316c | Kisanda | SubSaharan Africa 10 | KX570805 | 34.0589 | 1.04752 |
| Nakasongola | Kiimatwe | UG340b | Kisanda | SubSaharan Africa 10 | KX570806 | 33.09299 | 0.27852 |
| Nakasongola | Kiimatwe | UG267b | Kisanda | SubSaharan Africa 10 | KX570807 | 32.58043 | 2.00771 |
| Wakiso | Jungo | UG267c | Mululuza | SubSaharan Africa 10 | KX570808 | 32.58043 | 2.00771 |
| Wakiso | Jungo | UG166Nb | Mululuza | SubSaharan Africa 10 | KX570809 | 31.88285 | 0.84068 |
| Wakiso | Jungo | UG160c | Mululuza | SubSaharan Africa 10 | KX570810 | 31.50617 | 1.26125 |
| Bukedea | Bukedea | UG202b | Wandering Jew | SubSaharan Africa 10 | KX570811 | 32.30955 | 0.44537 |
| Bukedea | Bukedea | UG306c | Wandering Jew | SubSaharan Africa 10 | KX570812 | 34.09404 | 1.09966 |
| Bukedea | Bukedea | UG76c | Wandering Jew | SubSaharan Africa 10 | KX570813 | 32.34638 | 0.21412 |
| Kaberamaido | Ogasia | UG249ac | Wandering Jew | SubSaharan Africa 10 | KX570814 | 32.21359 | 2.13332 |
| Kaberamaido | Ogasia | UG146 | Wandering Jew | SubSaharan Africa 11 | KX570815 | 30.72763 | 0.76229 |
| Kagadi | Kibanga | UG152 | Wandering Jew | SubSaharan Africa 11 | KX570816 | 30.93841 | 1.07421 |
| Kibuku | Tirinyi | UG152b | Wandering Jew | SubSaharan Africa 11 | KX570817 | 30.93841 | 1.07421 |
| Kibuku | Tirinyi | UG304b | Wandering Jew | SubSaharan Africa 12 | KX570818 | 34.03827 | 1.18822 |
| Kyankwanzi | Kikolimbo | UG304 | Wandering Jew | SubSaharan Africa 12 | KX570819 | 34.03827 | 1.18822 |
| Kyankwanzi | Kikolimbo | UG304c | Wandering Jew | SubSaharan Africa 12 | KX570820 | 34.03827 | 1.18822 |
| Kyankwanzi | Kikolimbo | UG279 | Wandering Jew | SubSaharan Africa 12 | KX570821 | 33.13734 | 1.53665 |
| Masaka | Mwalo | UG279d | Wandering Jew | SubSaharan Africa 12 | KX570822 | 33.13734 | 1.53665 |
| Masaka | Mwalo | UG150cB | Wandering Jew | SubSaharan Africa 12 | KX570823 | 30.78187 | 0.92265 |
| Masaka | Mwalo | UG324 | Wandering Jew | SubSaharan Africa 12 | KX570824 | 33.46321 | 1.00456 |
| Ngora | Akeit | UG324 | Wandering Jew | SubSaharan Africa 12 | KX570825 | 33.46321 | 1.00456 |
| Ngora | Akeit | UG163 | Wandering Jew | SubSaharan Africa 12 | KX570826 | 31.7035 | 0.99178 |
| Ngora | Akeit | UG163b | Wandering Jew | SubSaharan Africa 12 | KX570827 | 31.7035 | 0.99178 |
| Oyam | Acero | UG163c | Wandering Jew | SubSaharan Africa 12 | KX570828 | 31.7035 | 0.99178 |

| **District** | **Village** | **Sample no.** | **Host plant** | **Whitefly species** | **Accession no.** | **Longitude** | **Latitude** |
| --- | --- | --- | --- | --- | --- | --- | --- |
| Luweero | Ngaju | UG211c | Wandering Jew | SubSaharan Africa 13 | KX570829 | 32.28148 | 1.00475 |
| Mpigi | Maziba | UG31 | Wandering Jew | SubSaharan Africa 12 | KX570830 | 31.70134 | -0.32107 |
| Kiboga | Kabindo | UG31b | Wandering Jew | SubSaharan Africa 12 | KX570831 | 31.70134 | -0.32107 |
| Luweero | Butanze | UG31c | Wandering Jew | SubSaharan Africa 12 | KX570832 | 31.70134 | -0.32107 |
| Ngora | Akeit | UG77bB | Kisanda | SubSaharan Africa 13 | KX570833 | 32.34638 | 0.21412 |
| Oyam | Acero | UG294 | Wandering Jew | SubSaharan Africa 12 | KX570834 | 33.49901 | 1.35491 |
| Luweero | Butanze | UG294b | Muwugula | SubSaharan Africa 12 | KX570835 | 33.49901 | 1.35491 |
| Soroti | Opiro | UG294c | Muwugula | SubSaharan Africa 12 | KX570836 | 33.49901 | 1.35491 |
| Soroti | Opiro | UG285c | Muwugula | SubSaharan Africa 12 | KX570837 | 33.39515 | 1.42681 |
| Wakiso | Bulamu | UG202d | Mululuza | Mediterranean | KX570838 | 32.30955 | 0.44537 |
| Wakiso | Bulamu | UG71 | Mululuza | Mediterranean | KX570839 | 32.34638 | 0.21412 |
| Kagadi | Kibanga | UG181 | Wandering Jew | Mediterranean | KX570840 | 32.28541 | 0.51368 |
| Kyenjojo | Mukole | UG181b | Muwugula | Mediterranean | KX570841 | 32.28541 | 0.51368 |
| Kyenjojo | Mukole | UG181c | Cassava | Mediterranean | KX570842 | 32.28541 | 0.51368 |
| Budaka | Gadomere | UG181d | Cassava | Mediterranean | KX570843 | 32.28541 | 0.51368 |
| Budaka | Gadomere | UG181e | Cassava | Mediterranean | KX570844 | 32.28541 | 0.51368 |
| Buyikwe | Naminya | UG166N | Cassava | SubSaharan Africa 2 | KX570845 | 31.88285 | 0.84068 |
| Dokolo | Bardyang | UG204d | Cassava | SubSaharan Africa 2 | KX570846 | 32.30955 | 0.44537 |
| Dokolo | Bardyang | UG293 | Cassava | SubSaharan Africa 2 | KX570847 | 33.49901 | 1.35491 |
| Kiboga | Kabindo | UG258 | Wandering Jew | SubSaharan Africa 2 | KX570848 | 32.38544 | 2.18531 |
| Kyankwanzi | Kijikwa | UG204 | Cassava | SubSaharan Africa 6 | KX570849 | 32.30955 | 0.44537 |
| Luweero | Butanze | UG290 | Mululuza | SubSaharan Africa 6 | KX570850 | 33.39515 | 1.42681 |
| Mbale | Fikasalama | UG290c | Cassava | SubSaharan Africa 6 | KX570851 | 33.39515 | 1.42681 |
| Mpigi | Mazibamutubagumu | UG364 | Kisanda | SubSaharan Africa 6 | KX570852 | 32.38472 | 0.26245 |
| Oyam | Arok | UG364b | Cassava | SubSaharan Africa 6 | KX570853 | 32.38472 | 0.26245 |
| Kibaale | Kilanza | UG150bS | Muwugula | SubSaharan Africa 9 | KX570854 | 30.78187 | 0.92265 |
| Kibaale | Kilanza | UG146bB | Muwugula | SubSaharan Africa 9 | KX570855 | 30.72763 | 0.76229 |
| Kyenjojo | Mukole | UG143b | Muwugula | SubSaharan Africa 9 | KX570856 | 30.68388 | 0.70078 |
| Kagadi | Kibanga | UG150 | Wandering Jew | Bemisia Uganda1 | KX570857 | 30.78187 | 0.92265 |
| Kiboga | Kabindo | UG166Nc | Wandering Jew | Bemisia Uganda1 | KX570858 | 31.88285 | 0.84068 |
| Kiboga | Sinde | UG170 | Joobyo | Bemisia Uganda1 | KX570859 | 31.94805 | 0.78855 |
| Kiboga | Sinde | UG170cS | Joobyo | Bemisia Uganda1 | KX570860 | 31.94805 | 0.78855 |
| Luweero | Bajjomituba | UG193 | Kisanda | Bemisia Uganda1 | KX570861 | 32.32081 | 0.33633 |
| Luweero | Bajjomituba | UG193c | Kisanda | Bemisia Uganda1 | KX570862 | 32.32081 | 0.33633 |
| Luweero | Bajjomituba | UG193d | Kisanda | Bemisia Uganda1 | KX570863 | 32.32081 | 0.33633 |
| Luweero | Ngaju | UG211 | Wandering Jew | Bemisia Uganda1 | KX570864 | 32.28148 | 1.00475 |
| Luweero | Ngaju | UG211b | Wandering Jew | Bemisia Uganda1 | KX570865 | 32.28148 | 1.00475 |
| Masaka | Bukona | UG21c | Muwugula | Bemisia Uganda1 | KX570866 | 31.66463 | -0.44107 |
| Masindi | Kisalabwire | UG231c | Mululuza | Bemisia Uganda1 | KX570867 | 31.4353 | 1.42726 |
| Masindi | Kisalabwire | UG231d | Mululuza | Bemisia Uganda1 | KX570868 | 31.4353 | 1.42726 |
| Wakiso | Butaku | UG178 | Joobyo | Bemisia Uganda1 | KX570869 | 32.28541 | 0.51368 |

**Supplementary Dataset:** *Bemisia tabaci* and non-*tabaci* species collected from cassava and six weed plant species from various locations during August–November 2013 in Uganda. Putative species were assigned based on their partial mtCO1 sequences according to Dinsdale *et al.* (2010).
